# The Polish face in profile: a cephalometric baseline study

**DOI:** 10.1186/s13005-015-0065-x

**Published:** 2015-03-19

**Authors:** Jolanta E Loster, Stephen Williams, Aneta Wieczorek, Bartłomiej W Loster

**Affiliations:** Department of Prosthodontics, Dental Institute, Faculty of Medicine, Jagiellonian University, Kraków, Poland; Department of Orthodontics, Dental Institute, Faculty of Medicine, Jagiellonian University, Kraków, Poland

## Abstract

**Background:**

This study reports the cephalometric evaluation of a group of adolescent Polish individuals describing dento-facial structure as well as details of incisor position and soft tissue characteristics. The results should reveal morphological features specific to Polish persons and serve as a comparative material for future diagnostic procedures.

**Methods:**

The study was based on an analysis of cephalgrams of 122 Polish adolescents average age 18years 6 months analysed in a computer system using the Kracovia composite system analysis describing dento-facial morphology ad modum Björk as well as soft tissue factors. The control material was based on published reports by Björk (Dento-facial characteristics) Riketts and Holdaway (soft tissue profile).

**Results:**

The comparative study revealed a slight reduction in the sagittal jaw relationship with a significant reduction in the vertical jaw relationship and a distinctive mandibular morphology with a reduced jaw angle and an increase in the "Beta angle". These findings were reflected in the soft tissue pattern. The soft tissue profile reflected the skeletal cephalometrics observation.

**Conclusion:**

The dento-facial profile of Polish adolescents demonstrates specific characteristics which should be taken into account when diagnosing facial form in connection with orthodontic treatment planning in particular Polish patients.

## Introduction

Despite many technical advances, the measurement of parameters on a two-dimensional cephalometric registration as introduced by Broadbent [1] remains an important and worthwhile element in orthodontic treatment planning. The results of the cephalometric analysis are usually expressed as angles, the value of which are often compared with tables representing average values for a given population. The aim is of course to localise possible morphological variations which could explain the biology of the given malocclusion. It is widely recognised that the value of cephalometric norms used for comparison are related to the nationality of the population on which the control material is based. Many studies have demonstrated clear morphological differences between dento-facial parameters of individuals of different ethnic background [2-4]. In describing European subjects it is quite customary to describe participants as “Caucasians”, a term which could give the impression of some degree of homogeneity whereas in fact a considerable degree of variation in anatomical morphology, including craniofacial form exists. Compared with many areas of Europe the population of Poland can be considered reasonably homogenic and consequently it can be considered worthwhile to evaluate cephalometric parameters describing the dento-facial skeleton of young Polish subjects with the intention of creating a cephalometric reference material specific for this nationality.

It is now generally accepted that facial aesthetics constitute a significant factor in the indication for orthodontic treatment which often involves orthognathic surgery in the correction of malocclusion [5]. Evaluation of the soft tissue profile, by comparison with average values of relevant parameters can be of great value though due to obvious racial differences evaluation must be compared with material based on similar ethic origins.

Estimation of the *sagittal jaw relationship* can in many ways be considered a basic step in the differential diagnosis of malocclusion and has been traditionally based on the ANB angle defined by Downs [6], later demonstrated by Jacobsen [7] to be unreliable due to geometric problems which has been shown could easily affect the interpretation [8]. A study comparing different parameters describing sagittal jaw relationship [9] found very little correlation between the parameters investigated.

Over many years the *position of the mandibular incisors* relative to the jaw base as well as maxillary incisors has formed an important step in orthodontic treatment planning and various norms have been described based on theories regarding stability [10] as well as function [11,12]. Despite many years of study of incisal position no well-substantiated guidelines really exist and an investigation based on the present untreated sample of Polish juveniles affords the opportunity to investigate *natural incisor orientation* in relatively harmonious occlusion.

The aim of the study was to investigate the dento-facial morphology and soft tissue characteristics of a group of young adolescent individuals of Polish nationality by means of cephalometrics, thus establishing a series of values (norms) which can serve as a comparative material for future orthodontic treatment of Polish patients. The variation in incisal orientation (angular inclination and linear protrusion) was been investigated. Using the opportunity provided by the extensive nature of the study material the sagittal skeletal jaw relationship evaluated by a number of methods will also be reported and compared.

This study was approved by the bioethical committee of the Jagiellonian University, Krakow with the approval certificate number KBET/89/B/2009.

## Method and materials

The present study is based on standardised digital cephalometric recordings (low dosage protocol) of 122 individuals (35 males, 87 females) with an average age of 18 years six months, minimum 17 years 2 months and maximum 19 years 7 months. Material was collected in connection with a larger study investigating function of the stomatognathic system in the Polish population. All the participants fulfilled the Research Diagnostic Criteria for Temporo Mandibular Disorders Polish Version Questionnaire (RDC/TMD) and in point 25 axis II they described their Polish nationality [13]. None of the participants included in the study had received orthodontic treatment and were included irrespective of occlusion. The study was performed using the statistical premise used by Björk and internationally accepted since 1947. Since the number of subjects was large it was considered acceptable to base results on the entire material, irrespective of occlusion/malocclusion. The participants and their parents were informed about examination procedure and both of them had signed informed consent form. The study was conducted in accordance with the Declaration of Helsinki and Good Clinical Practise (GCP) rules. All cephalograms in the study material were recorded under standardized conditions on the same machine (Planmeca ProMax, Helsinki, Finland 2005) and measurement was performed by the same observer (BWL) using a FACAD© computer cephalometric system (Ilexis AB, Sweden 2014) which simultaneously calibrated all radiographs to the same enlargement factor. All data was calculated using the statistical facility in an Excel® spreadsheet.

### The basic analysis

The Kracovia composite analysis [14] consists basically in three parts, the first being the full cephalometric analysis as outlined by Björk [15] and further elaborated in a Scandinavian textbook [16]. This analysis describes the occlusion in a unique way, differentiating between skeletal, alveolar and dental characteristics in the sagittal and vertical planes, as well as describing mandibular morphology and the flexure of the cranial base. The concept of skeletal and dento-alveolar components and the principles of compensation and dysplasia were further outlined and elaborated by Solow [17].

**Figure 1:**
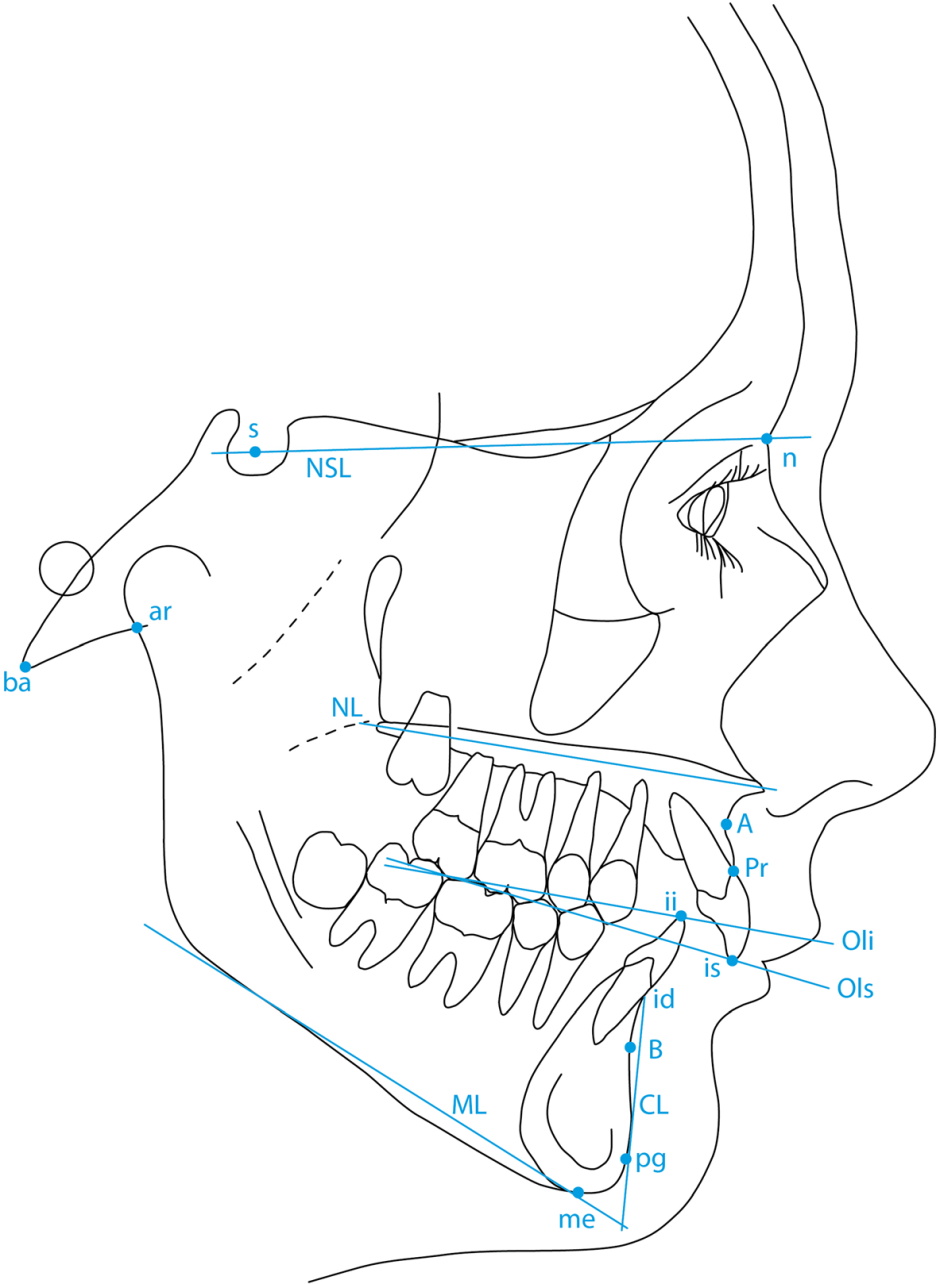
**The cephalometric reference points used in the present study (Björk analysis).**

The reference points used in this study are illustrated in Figure 1 and include the following:

- s - Sella. The midpoint of sella turcica.
- n - Nasion. The most anterior point of the fronto-nasal suture.
- ar - Articulare. The point of intersection of the contour of the external cranial base and dorsal contour of the condylar process.
- ba - Basion. The lowest, most anterior point on the clivus and median point on the anterior border of the foramen magnum.
- A- Downs A point. The deepest point of the anterior curvature of the maxillary alveolar process in the midline (point *subspinale (ss)* in Björk).
- pr - Prosthion. The lowest and most anterior point on the surface of the maxillary alveolar process.
- id - Infradentale. The highest and most anterior point of the mandibular alveolar process.
- B - Downs B point. The deepest point on anterior curvature of the alveolar process of the mandible (point *supramentale* (sm) in Björk).
- pg - Pogonion. The most anterior point of the anterior surface on the bony chin.
- me - Menton (named *gnathion* in Björk). The lowest point on the mandibular symphysis measured from nasion.
- is - incision superius. The middle of the incisal edge of the most prominent maxillar incisor
- ii - incision inferius. The tip of the most prominent mandibular incisor.

Figure 1 also illustrates the following reference lines (All from Björk [15]).

- NSL Sella Nasion line
- NL Nasal line (anterior nasal spine – posterior nasal spine)
- OLs Maxillary occlusal plane (tip of disto buccal cusp of the maxillary first molar to incision superius)
- OLi Mandibular occlusal plane (tip of disto buccal cusp of the first molar to incision inferius)
- ML Mandibular line. A tangent to the lower border of the mandible with the origin through menton.
- CL Chin line. A line tangent to the anterior border of the mandibular symphysis from point id

**Figure 2:**
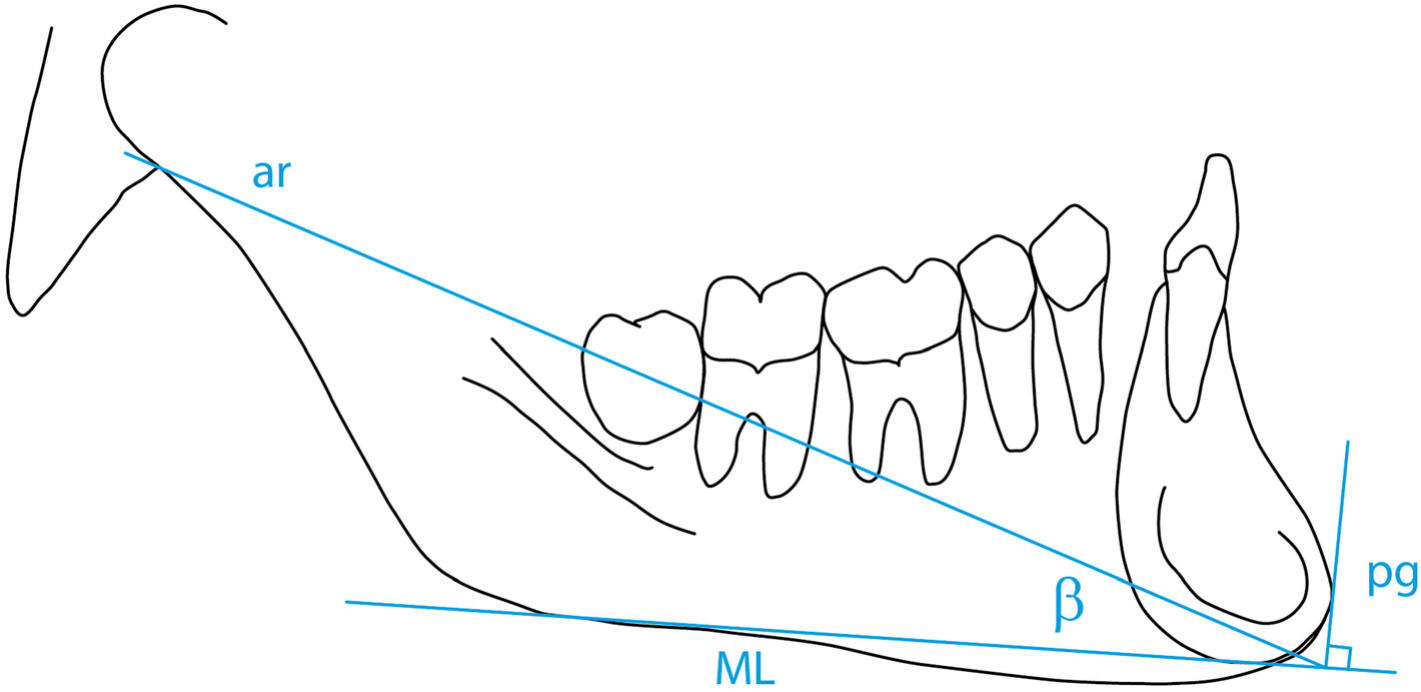
**The construction of the β angle: a line from the point articulare to the point of origin of a line tangent to the anterior border of the symphysis and perpendicular to the mandibular line.** The β angle is created between this constructed line and the ML.

The parameters derived from the reference points above are listed in Table 1 and illustrated in Figure 1 and describe skeletal jaw relationships sagittal (1–5) and vertical (11–13) as well as the dento-alveolar relationships in the sagittal (6–10) and vertical planes (14–15). The analysis also describes the shape of the mandible expressed as the β angle illustrated in Figure 2, an angle which, in reality describes the relative height of the ramus and length of the ramus.

**Table 1:** **Comparison of results of the cephalometric analysis of skeletal, dento-alveolar and soft tissue factors of the Polish study material compared to the control group (Björk)**

	**Cephalometric table**		**mean**	**SD/Range**	**Polish average**	**Polish SD**	**T value**	**sign**
	**Björk**		**N = 320**		**N = 122**			
	**Sagittal jaw relationship**							
1	A-N-pg	A-N-pg	2.0°	2.5°	1.31°	2.86°	1.093	n.s.
2	A-N-B	A-N-B	3.0°	2.5°	2.56°	2.44°	0.733	n.s.
	**Jaw prognathism**							
3	Maxillary (A)	S-N-A	82.0°	3.5°	81.72°	3.69°	0.385	n.s.
4	Mandibular (pg)	S-N-pg	80.0°	3.5°	80.42°	4.00°	0.562	n.s.
5	Mandibular (B)	S-N-B	79.0°	3.0°	79.16°	3.80°	0.224	n.s.
	**Dento-alveolar prognathism**							
6	Maxillary	pr-N-A	2.0°	1.0°	2.11°	1.03°	0.285	n.s.
7	Mandibular (CL)	CL/ML	70.0°	6.0°	70.88°	6.25°	0.927	n.s.
8	Mandibular (B)	pg-N-B	1.0°	2.5°	1.25°	0.88°	0.534	n.s.
	**Incisor inclination/base**							
9	Maxillary	Ils/NL	110.0°	6.0°	112.10°	7.13°	2.120	*
10	Mandibular	Ili/ML	94.0°	7.0°	94.00°	7.25°	0.000	n.s.
	**Vertical relationship**							
11	Vertical jaw relationship	NL/ML	25.0°	6.0°	21.18°	5.81°	4.116	***
12	Maxillary inclination	NL/NSL	8.0°	3.0°	8.88°	2.92°	1.339	n.s.
13	Mandibular inclination	ML/NSL	33.0°	6.0°	30.05°	6.08°	3.134	**
	**Vertical dento-alveolar condition**							
14	Maxillary zone	NL/OLs	10.0°	4.0°	7.17°	3.92°	3.721	***
15	Mandibular zone	OLi/ML	20.0°	5.0°	18.13°	3.76°	2.375	**
	**Mandibular morphology**							
16	Beta angle		19.0°	2.5°	22.13°	2.94°	4.913	***
17	Jaw angle		126.0°	6.0°	121.64°	6.70°	4.492	***
	**Cranial base**							
18	N-S-ar		124.0°	5.0°	123.67°	5.74°	0.369	n.s.
19	N-S-ba		131.0°	4.5°	130.85°	5.23°	0.176	n.s.

*p < 0.05.

**p < 0.01.

***p < 0.001.

### The sagittal jaw relationship and incisal position

**Figure 3:**
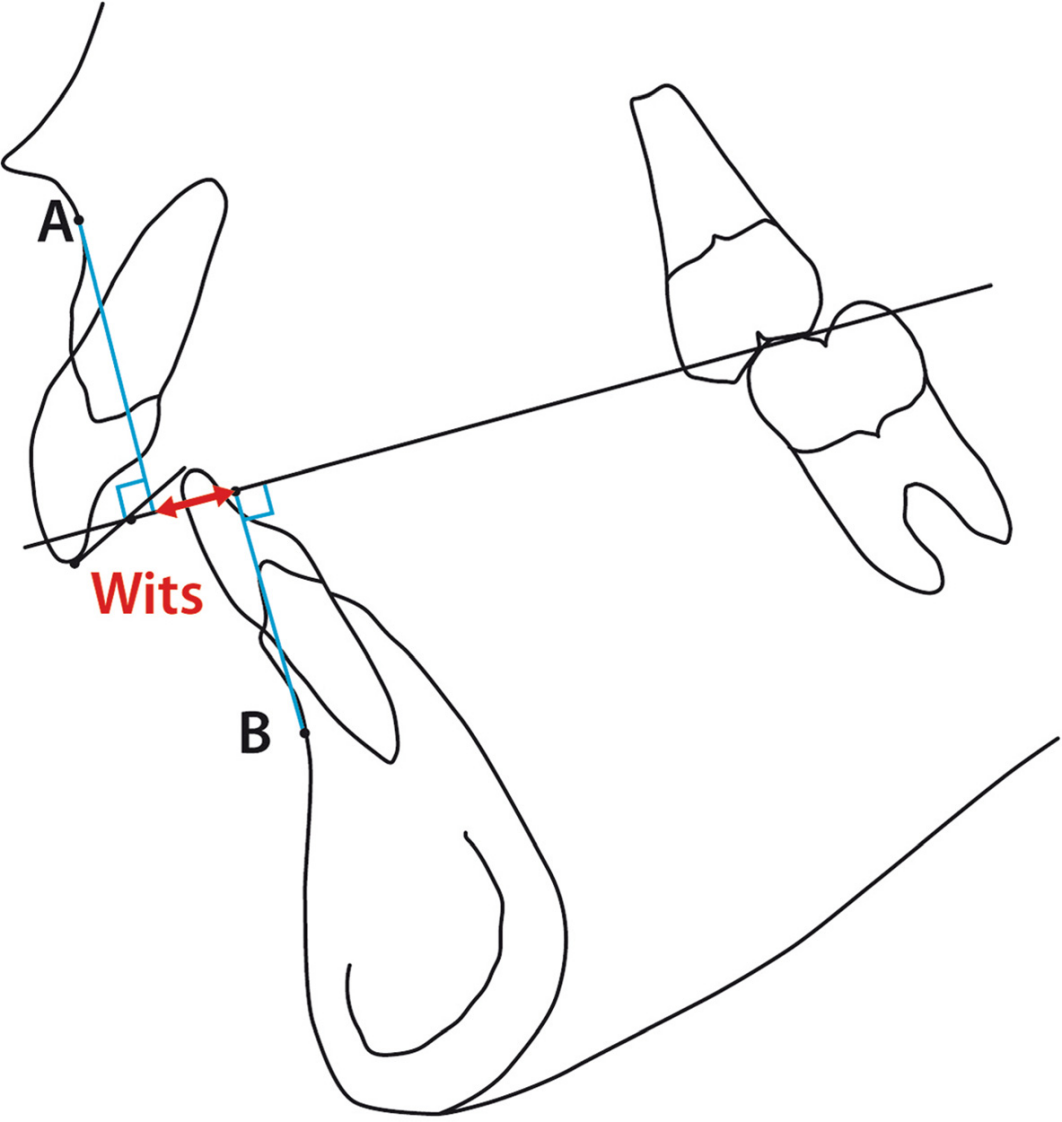
**The Wits analysis.**

**Figure 4:**
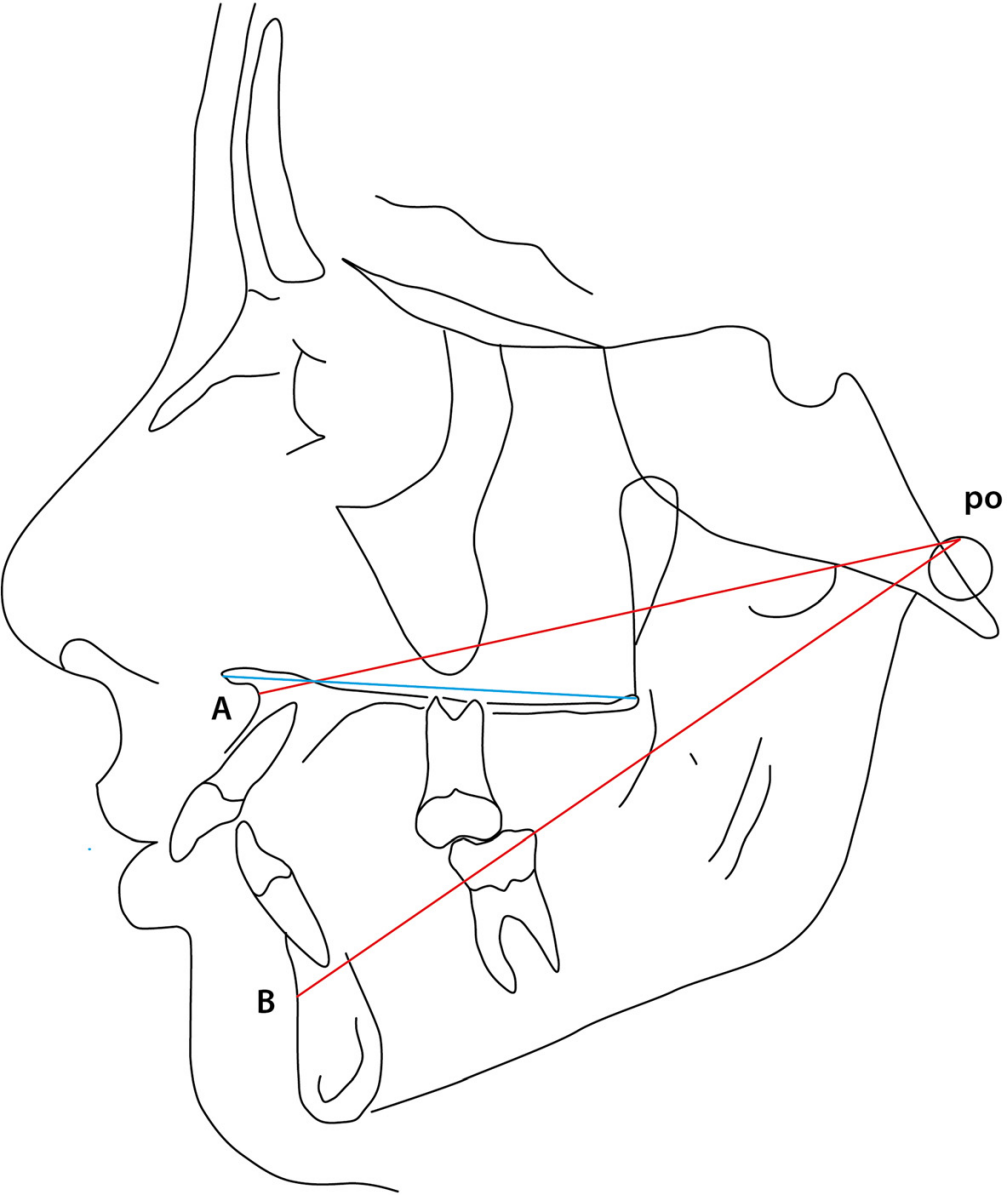
**Linear expression maxillary and mandibular length/prognathism relative to the point porion (po) (var. 21, 22 see Table** 2 **)**.

The second part of the analysis considers the sagittal jaw relationship. Due to doubts concerning the value of the ANB angle as suggested by Jacobsen [7] the linear “Wits Analysis” is also included in part 2 of the cephalometric analysis (var. 20 (Table 2) and Figure 3). Since also the value of the Wits analysis has been drawn into doubt [8] a third evaluation of the sagittal jaw relationship was considered based on the length/ protrusion of the maxilla and mandible (as well as their relative lengths) related to the point Porion (po), replicating a system devised by Harvold [18] though purposely avoiding the use of the point *articulare*. The parameters are illustrated in Figure 4 and represented by variables 21, 22, 23 in Table 2.

**Table 2:** **Comparison of results of the cephalometric analysis of skeletal, dento-alveolar and soft tissue factors of the Polish study material compared to the control group (Björk)**

	**Supplementary values**					
	**Wits appraisal**		**Mean Björk**	**SD/Range**	**Polish average**	**Polish SD**
20	Wits		0.0mm		0.70mm	3.14mm
	**Jaw lengths (to Porion)**					
21	Maxilla	Po-A	mm		89.62mm	5.34mm
22	Mandible	Po-B	mm		102.14mm	6.29mm
23	Relative jaw length		%		87.79	3.09
	**Incisal inclination to OP**					
24	Maxillary	Ils/OP	60.7°		63.18°	6.26°
25	Mandibular	Ili/OP	72.0°		69.55°	6.67°
26	Inter-incisal angle		132.0°		132.73°	10.09°
	**Incisal relationship**					
27	Maxillary incisor proclination	Iis/NA			21.50^o^	7.47°
28	Maxillary incisor protrusion	Is-NA			3.04^o^	2.49°
29	Mandibular incisor proclination	Ili/NB			23.21^o^	6.05°
30	Mandibular incisor protrusion	Ii-NB			3.29^o^	2.17°
31	ii to A-pg		1.0 mm		0.85mm	2.22mm

### Incisal position and relationship

**Figure 5:**
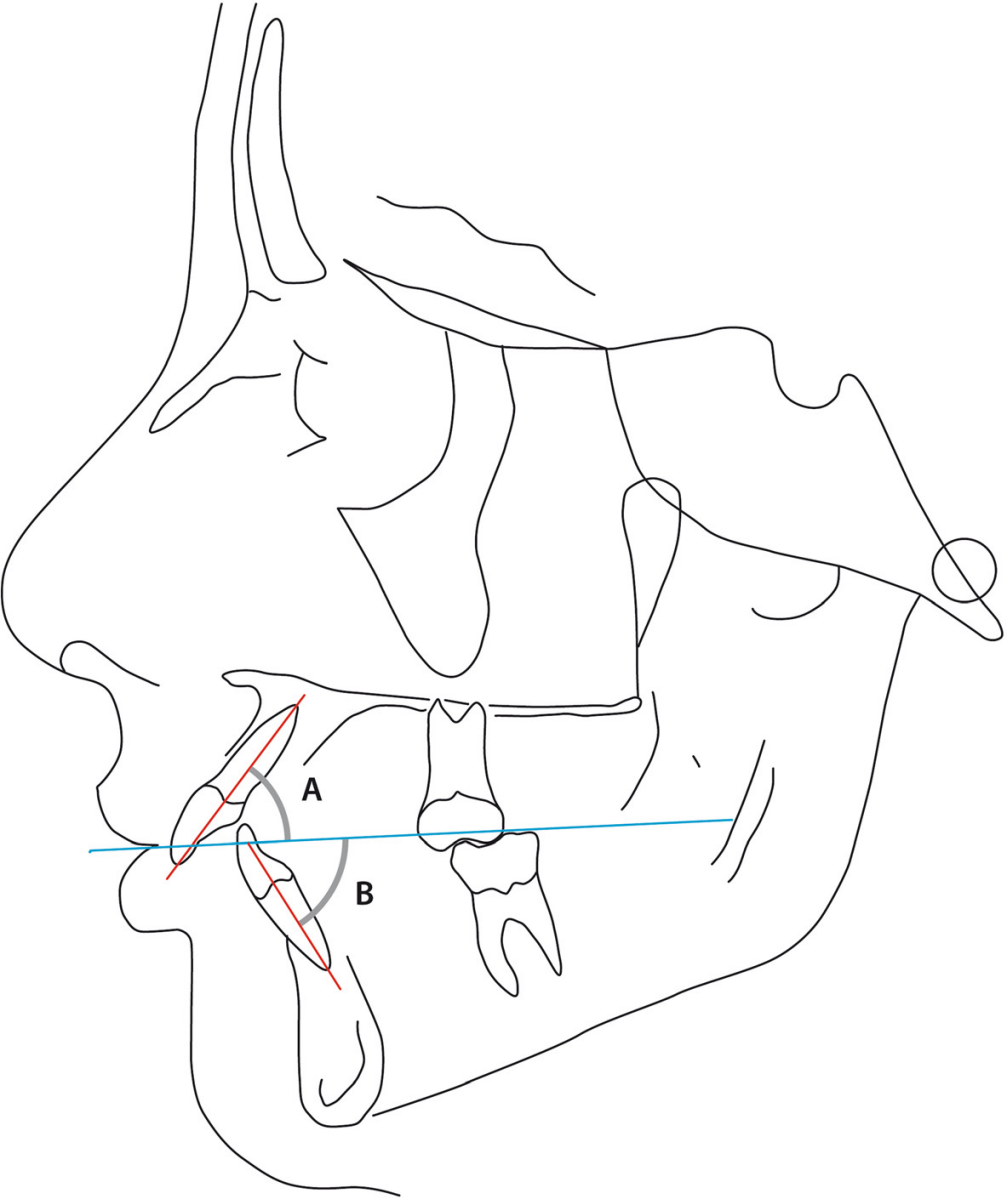
**The angulation of the maxillary incisors (angle A var. 24) and mandibular incisors (B, var. 25) and inter-incisal angle (A + B var. 26).**

**Figure 6:**
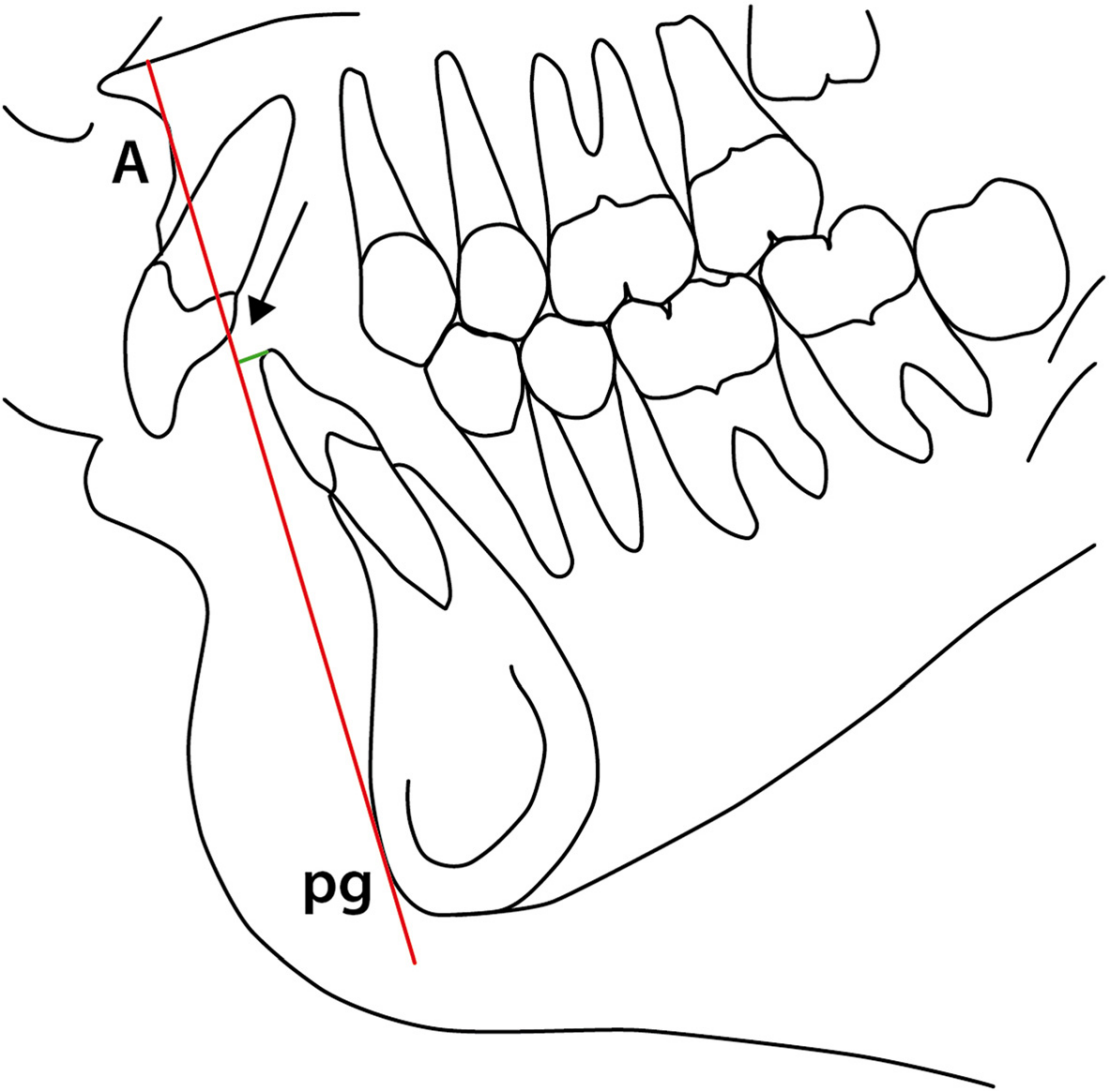
**Protrusion of lower incisor to A-pg line (Ricketts** [21]**) (var. 31 see Table** 2 **).**

The inclination of the maxillary and mandibular incisors was estimated relative to the occlusal plane (var. 24 and 25 Table 2 and Figure 5) as was the inter-incisal angle (var. 26). The protrusion of the incisors, maxillary and mandibular was also measured as an angle and a linear distance to the NA and NB lines respectively as described by Steiner [19,20]. Finally the protrusion of the mandibular incisors as described by Ricketts [21] relative to the A-pg line was recorded (Figure 6).

### The soft tissue profile

**Figure 7:**
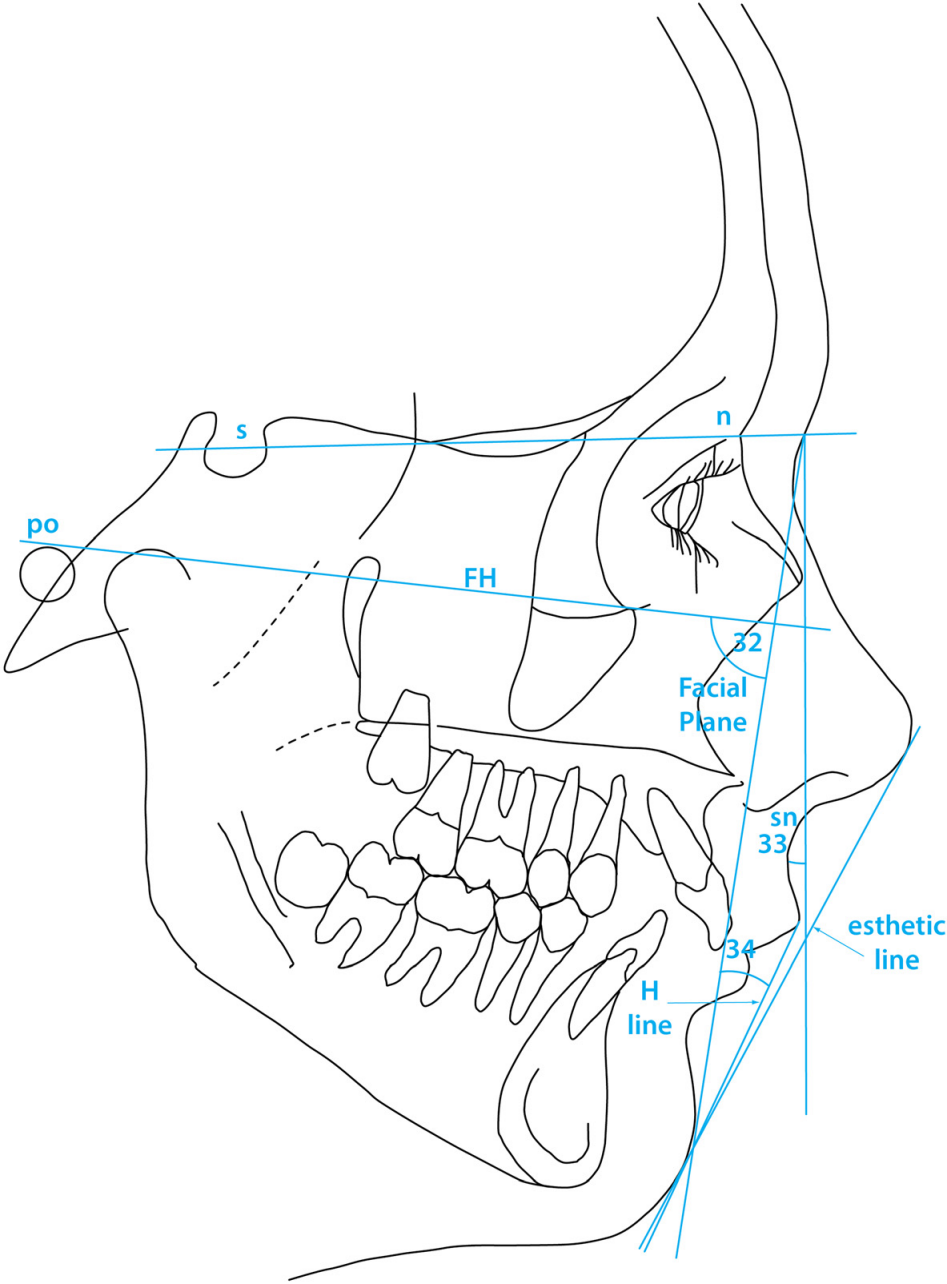
**Facial planes used in the present study as well as the two angles used to describe chin prognathism (according to Holdaway** [22,23]**).**

**Figure 8:**
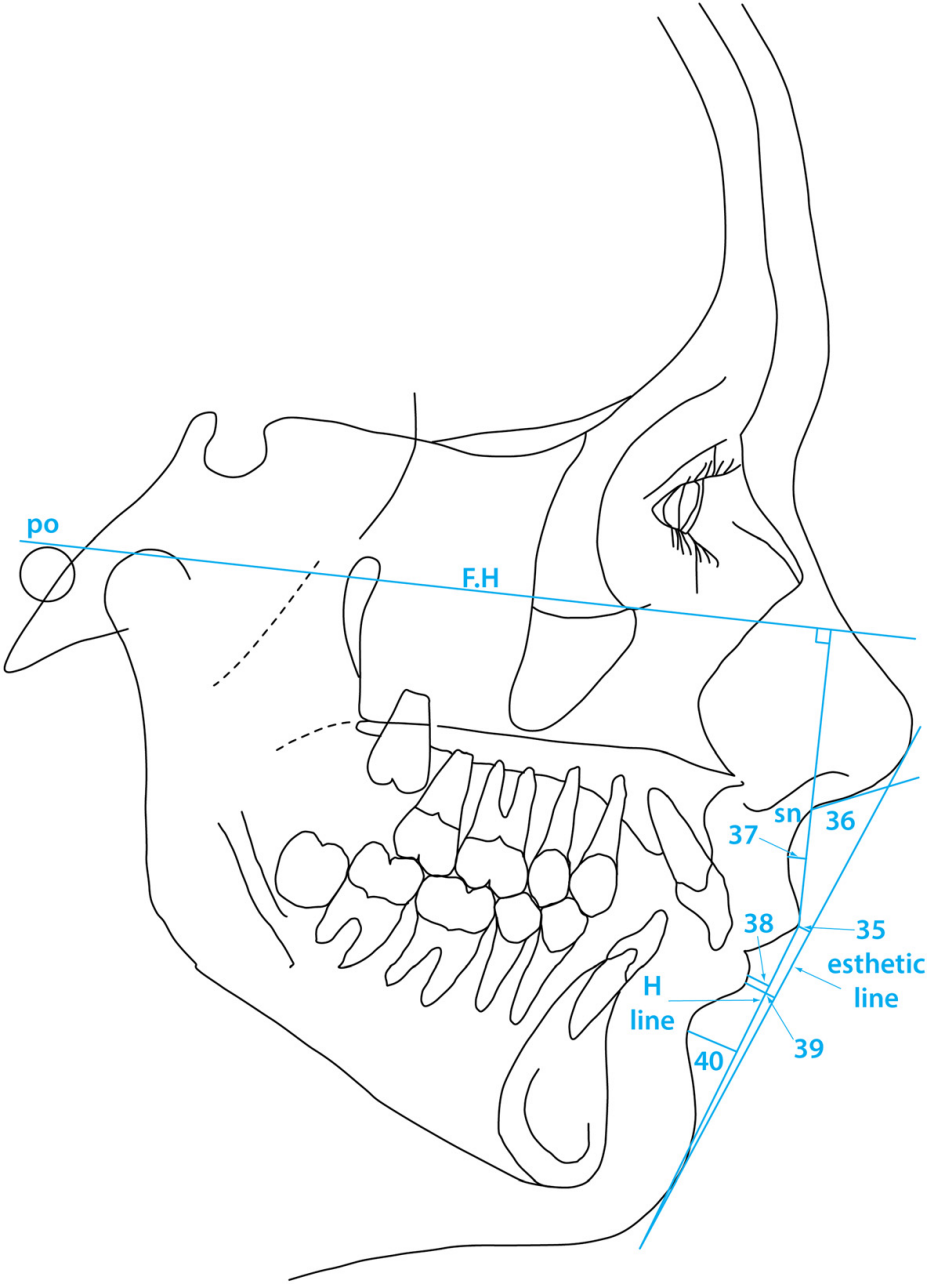
**Angles and linear measurements used to describe lip position and profile (Holdaway [** 22 **,** 23 **]).** The numbers shown can be interpreted by means of Table 3.

**Figure 9:**
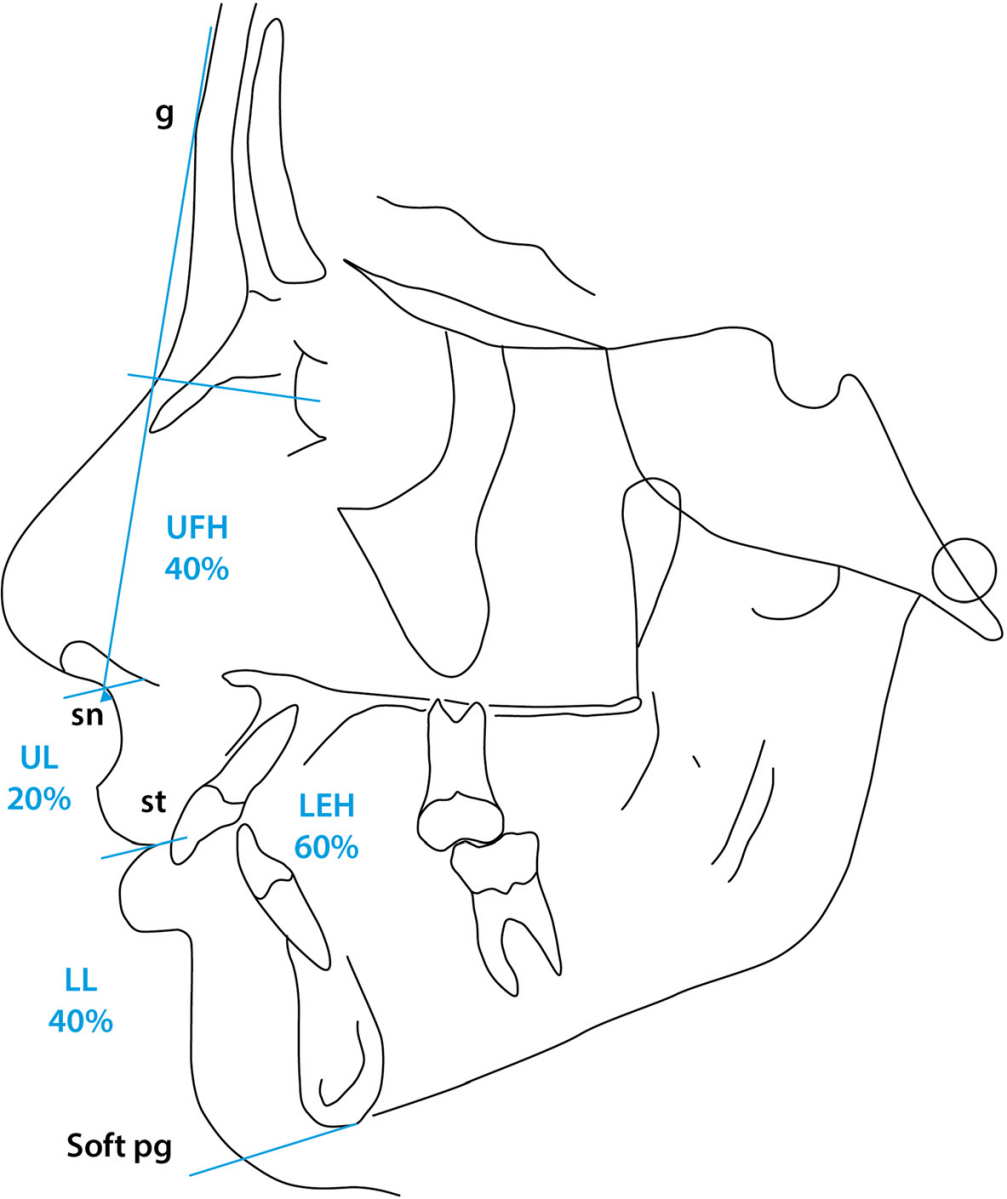
**Soft tissue proportions** ***ad modum*** **Isaacson** [5]**.**

The third part of the analysis describes the soft tissue profile as originally outlined by Holdaway [22,23], the parameters measured being shown in Table 3 and are illustrated in Figures 7, 8 and 9 (mean values and possible variation, where available, are quoted in Table 3 with the appropriate references to the source of the information).

**Table 3:** **Comparison of results of the cephalometric analysis of skeletal, dento-alveolar and soft tissue factors of the Polish study material compared to the control group (Björk)**

	**Soft tissue profile**	**Mean Björk**	**SD/Range**	**Polish average**	**Polish SD**
	Chin				
32	Soft tissue Facial Angle	91°	84°-98°	88.34°	4.58°
33	Facial Contour Angle	−11°	−7°-- 15°	−13.70°	5.87°
	Upper Lip				
34	Prominence (H Angle)		7°-14°	13.73°	4.71°
35	Lip tip to aesthetic Line	−4.0 mm		−5.01mm	2.18mm
36	Naso-labial Angle	110°		109.93°	10.72°
37	Depth of superior labial sulcus		−1.0- -4.0mm	−2.02mm	1.19mm
	Lower Lip				
38	Prominence (to H Line)	−0.5 mm	0.0 - 0.5 mm	0.08mm	1.63mm
39	Lip tip to aesthetic Line	−2.0 mm		−3.06mm	2.51mm
40	Depth of inferior labial sulcus	5.0 mm		−5.03mm	1.25mm
41	Depth of inferior labial sulcus (to H line)			−4.99mm	1.73mm
42	Lower lip/mandibular plane			50.87	6.44
	Vertical Dimensions				
43	Upper Facial Height (UFH)	40%		42.06%	2.95
44	Lower Facial Height (LFH)	60%		57.94%	2.95
45	Upper Lip (UL)	20%		18.51%	2.08
46	Lower Lip (LL)	40%		39.42%	2.51

### The sagittal jaw relationship and association between selected morphological parameters

In a supplementary study based on the same material a correlation analysis was performed using selected parameters, the aim being:

1. to compare the results of three different measurements describing sagittal jaw relationship in order to compare conclusions.
2. to investigate the correlation between the flexure of the cranial base and jaw prognathism and vertical skeletal conditions.

## Results

Considering the general cephalometric analysis the information derived from the Polish material compared with the findings of Björk revealed some similarity though also statistically significant differences in some parameters. Table 1, 2 and 3 can be summarised as follows (figures in parenthesis refer to the appropriate line in Tables 1, 2 and 3).

**Figure 10:**
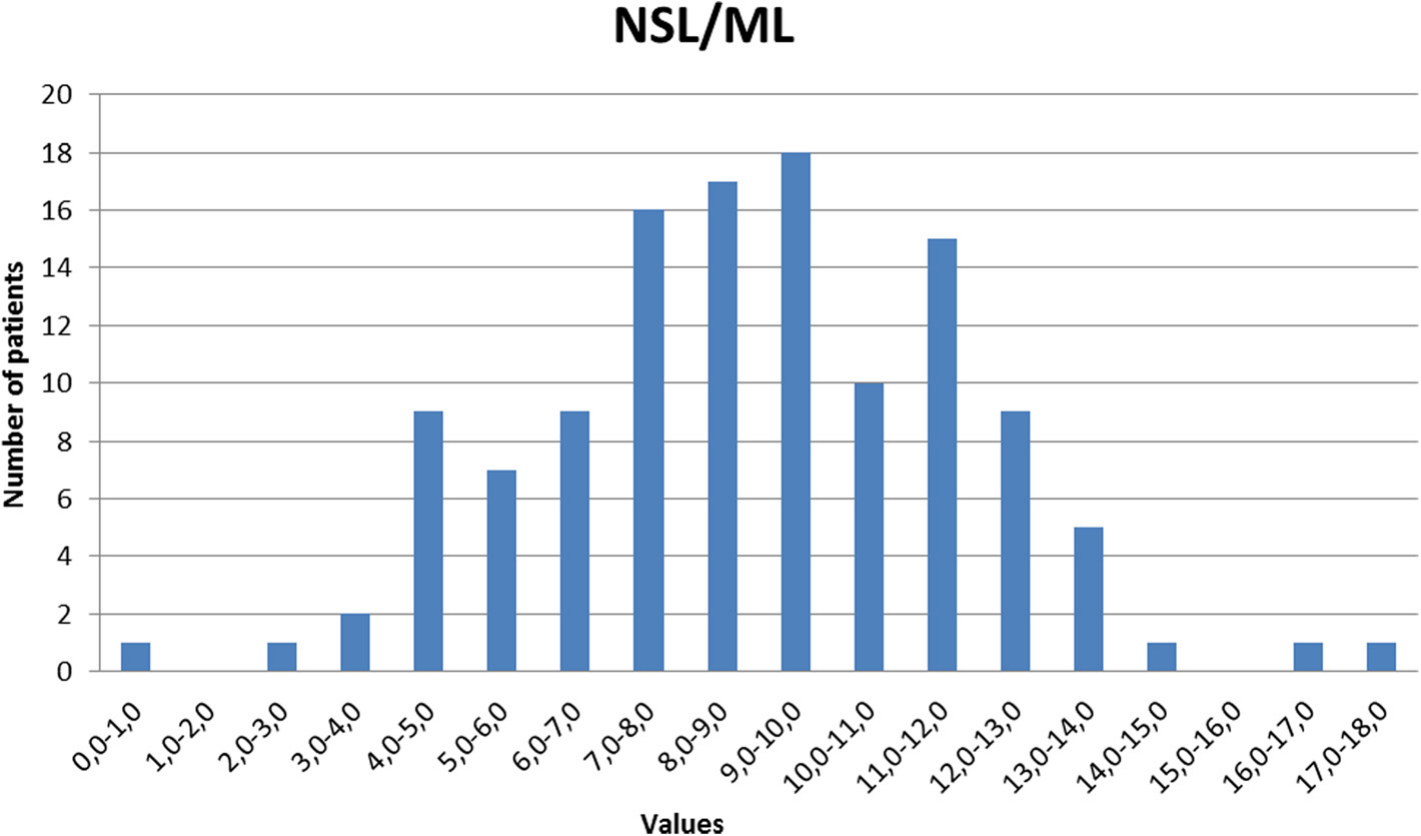
**Distribution of the vertical jaw relationship, angle NL/ML (variable 11) for the Polish study material.**

A slightly decreased sagittal relationship in the Polish material (var. 1, 1.31° t = 2.345, p < 0.01 and var. 2, 2.56° t = 1.683, p < 0.05). A single dental parameter reveals a slight statistical difference, namely the protrusion of the maxillary incisor to the skeletal base ILs/NL (var. 9) which is 2.1° larger in the Polish material (t = 2.886 p < 0.01). In the vertical plane significant differences were observed between the two groups, the skeletal vertical relationship (var. 11) of the Polish group (mean = 21.18°) being 3.82° less than the corresponding parameter in Björk’s Scandinavian material (mean = 25.0°) (t = 6.123, p < 0.001), due to a slight increase in posterior maxillary inclination in the Polish group (var. 12, t = 2.810, p < 0.01) as well as a clear anterior inclination of the mandible (var. 13) of 30.05° relative to the cranial base, t = 4.576, p < 0.001). This difference in vertical jaw relationship was accommodated by a compensatory reduction in both the maxillary zone (var. 14, 7.17°, t = 6.746, p < 0.001) and mandibular zone (variable 15, 18.13° t = 4.245, p < 0.001). A histogram demonstrating the distribution of the vertical jaw relationship (var. 11) is shown in Figure 10 and demonstrates a tendency to platykurtotic distribution representing the inclination of the mandible to the cranial base NSL/ML. The histogram demonstrates however a very broad distribution which is also represented in the size of the standard deviation of the two parameters are very similar to that seen in the control material.

Comparing the shape of the mandible between the two groups, based on the “beta” angle, representing the height of the mandibular ramus (var. 16) and the jaw angle (var. 17) revealed a clear inter-group difference, with the Polish group demonstrating a significantly larger beta angle (t = 10.411, p < 0.001) and reduced jaw angle (t = 6.290, p < 0.001). A comparison of the degree of flexure of the cranial base, based on group means and considered both medially (variable 19) and laterally (variable 18) demonstrate similarity in the two groups.

Concerning the supplementary values investigated (Table 2) it can be stated that the average relative jaw length ratio (maxilla to mandible, var. 23) was 87.79% with a standard deviation of 3.09%. No statistical comparison could be made with the control material, since these parameters are not included in the Björk study. The maxillary and mandibular incisor inclination and position relative to the NA, or NB line respectively (var. 27–30), correspond closely to the values suggested by Steiner [19,20]. Considering the inclination of the incisors to the occlusal plane (var. 24 and 25) the maxillary incisors were slightly retracted and thus more steep and the mandibular incisors slightly more proclined when compared with the variables by Riolo et al. [24] based on an American material, though again a statistical comparison was not possible.

**Figure 11:**
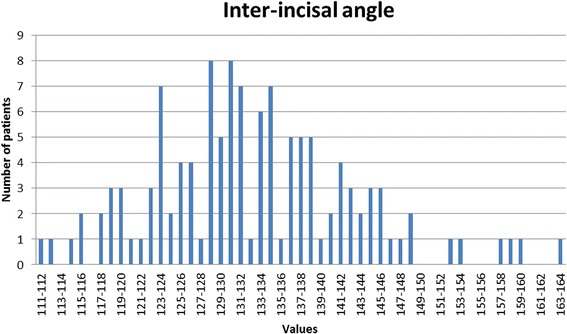
**Distribution of the inter-incisal angle as observed in the Polish material.**

**Figure 12:**
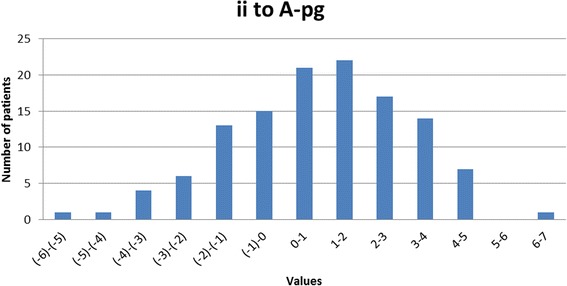
**Distribution of the protrusion of the mandibular incisors as expressed by the distance ii to a-pg line (var 31).**

**Figure 13:**
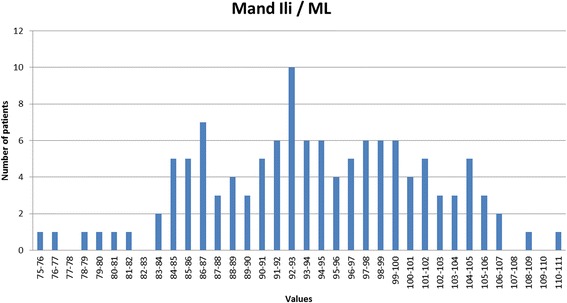
**The distribution of the inclination of the mandibular incisor relative to the mandibular base (var.10) observed in the Polish study material.**

The inter-incisor angle (var. 26) was on average 132.73° corresponding closely to the figures published by Steiner [19,20], though the standard deviation of 10.9° must be remembered. A histogram demonstrating the distribution of the inter-incisal angle is presented as Figure 11 demonstrating a normal distribution though with a range from 111° to 163°. The position of the lower incisal edge (variable 27) showed a mean value of 0.85mm with a relatively small standard deviation (2.22mm corresponding closely to the figures described by Ricketts [21]. Again the histogram describing the distribution of values for this parameter (Figure 12) reveals a normal distribution. The angle of the mandibular incisor to the mandibular base (var 10) was on average 94.0° though with a standard deviation of 6.25°, which is clearly seen in the histogram Figure 13 which also reveals a range from 75° to 110°.

Considering the soft tissue profile (Figure 7), both the soft tissue facial angle (var. 32) and the facial contour angle (var. 33) demonstrated that the soft tissue chin in the Polish material was slightly recessive compared with the values described by Holdaway [22,23], though no statistical comparison was possible due to lack of control material information. The standard deviation for each parameter describing soft tissue profile is reported in the present report.

In keeping with the differences in the vertical skeletal dimensions the distribution of the vertical facial height was seen to differ slightly in the Polish material (Table 3) in that the upper facial height U.F.H. was relatively greater (42.06%) than that quoted as average [5] and correspondingly the lower facial height was reduced at 57.94% basically due to a slightly lower value of the distance from the subnasion to the stomion, (UL) (18.51%) in the Polish material against an average of 20% as quoted by Holdaway [22,23].

Considering the correlation analysis of different methods of describing the sagittal jaw relationship it will be seen in Table 4 that the correlation between the ANB and parameters representing the mandible are relatively high (SNB r = −0.36 p < 0.01 and SNpg r = −0.42 p < 0.001) as was the relationship between ANB and the Wits analysis (r = 0.74p < 0.001). The correlation between the relative Jaw relationship (linear evaluation from Po) to the Wits analysis was also very high (r = 0.81 p < 0.001) as it also was to the ANB (r = 0.70 p < 0.001) Interestingly, in the same way that the angular prognathism of the two jaws, expressed by SNA and SNB were highly correlated (r = 0.79 p < 0.001) as was the correlation between the linear parameters designed to express the sagittal position of the jaws (Po-A/Po-B r = 0.83 p < 0.001).

**Table 4:** **The results of the correlation analysis (Pearson) based on a number of parameters chosen to represent skeletal and cranial sagittal relationships**

	**A-N-pg**	**A-N-B**	**S-N-A**	**S-N-pg**	**S-N-B**	**ML/NL**	**ML/NSL**	**N-S-ar**	**N-S-ba**	**Wits**	**Po-A**	**Po-B**	**Rel. Jaw**	**ANS-PNS**
A-N-pg	1													
A-N-B	0.96***	1**												
S-N-A	0.27**	0.29***	1											
S-N-pg	−0.46***	−0.42**	0.73***	1										
S-N-B	−0.35**	−0.36**	0.79***	0.98***	1									
ML/NL	0.38**	0.31**	−0.18	−0.44**	−0.37**	1								
ML/NSL	0.39**	0.31**	−0.38**	−0.64***	−0.57***	0.88***	1							
N-S-ar	−0.09	−0.11	−0.44***	−0.34**	−0.36**	−0.13	0.07	1						
N-S-ba	0.01	−0.01	−0.41***	−0.38**	−0.39**	−0.04	0.16	0.89***	1					
Wits	0.63***	0.74***	0.15	−0.31**	−0.33**	−0.01	−0.09	−0.07	−0.03	1				
Po-A	−0.02	0.02	0.16	−0.16	0.14	−0.32**	−0.33**	0.17	0.06	0.19*	1			
Po-B	−0.36**	−0.38**	0.04	0.29*	0.29**	−0.21*	−0.21*	0.18	0.06	−0.29**	0.83***	1		
Rel. Jaw	0.59***	0.70***	0.20**	−0.24*	−0.26**	−0.18	−0.21*	−0.04	−0.01	0.81***	0.25*	−0.33**	1	
ANS-PNS	−0.01	0.06	0.15	0.14	0.10	−0.22	−0.26	−0.03	−0.08	0.25	0.57	0.42	0.24	1

*p < 0.05.

**p < 0.01.

***p < 0.001.

In general the sagittal position of the mandible expressed by SNpg or SNB correlated negatively to an increase in posterior inclination of the mandible ML/NSL (r = −0.64 and −0.57 respectively), both being significant at the p < 0.001 level).

Considering the relationship between the flexure of the cranial base (N-S-ar and N-S-ba) the results demonstrated a clear negative correlation to parameters expressing sagittal jaw prognathism *viz*: N-S-ba to SNA, r = −0.41p < 0. 01 and N-S-ba to SNB r = 0.039 p < 0.01.

## Discussion

Evidence from other studies indicates that for purposes of comparison of individual patient data with orthodontic cephalometric reference material (cephalometric norms) the latter must be based on material as ethnically homogenous as possible since differences in the morphology of individuals as a result of ethnic origin which will be reflected in the value of cephalometric norms [2-4].

Considering the European situation it is not unusual that the reference material with which European patients are compared is often from another part of the world, frequently USA as is the case where the widespread use of analyses by Downs [5], Sassouni [25], Steiner [19], Ricketts [21] etc. Europe is also represented by the analyses defined by Harvold [18], Hasund [26] and Björk [15]. No analysis based on patients of Polish origin have been recorded in the literature making an investigation of the craniofacial morphology of Polish individuals highly relevant.

In the creation of this study it was considered that the Björk cephalometric analysis should form the basis of the study since this is the one method which considers skeletal (basal) alveolar and dental components in order to explain the morphology of any occlusion in the sagittal and vertical planes as described by Solow [17]. The Björk philosophy encompassed also two other hypotheses, namely that the growth pattern of the mandible, reflected in the morphology of the mandible (beta angle) as well as the importance of the flexure of the cranial base. The latter, being unchangeable by orthodontic means should be considered a dominant feature in occlusal development. The material for the original Björk study was based on Swedish individuals and even considering the geographical distance close proximity to Poland to Sweden differences in ethnic origin make differences in occlusal and dental facial characteristics likely. The findings of the present study demonstrate that certain important differences between the two groups could be identified.

Concerning the standard cephalometric analysis *ad modum Björk* the principal differences were found to be in the vertical plane such that the Polish material represented the lower vertical jaw relationship related to an increased anterior inclination of the mandible. Considering the dental alveolar compensation, which is a unique feature of the Björk analysis, a significant reduction in both maxillary and mandibular zones was observed. A clear difference in mandibular morphology could be seen between the two groups such that the increased “beta” angle as defined by Björk [15] combined with a reduced jaw angle (gonion angle) illustrates a relatively “square” mandibular morphology in the Polish group, probably suggesting a history of an “anterior rotational” growth pattern in a large number of cases, a factor which would have clinical significance.

Relating general jaw prognathism, both maxillary and mandibular, the hypotheses of Björk [27] that jaw prognathism can be related to flexure of the cranial base seems to be supported by the present study, confirming also the correlation findings by Solow [28].

The principal findings of the supplementary study concerning the position of the incisors both relative as the inter-incisor angle and also to the occlusal plane revealed values similar to those reported by Tweed [10] Steiner [19] and Riolo [24] though the principal finding of the present study underlines the variability of these parameters such that a simple distance or angulation for every patient in connection with treatment planning is unrealistic. This seems to indicate that the relationship between incisors is not a purely an anatomical factor which can be predicted cephalometrically but could well be related to other external factors as for example the inclination of the articular plane in the temporomandibular joint as suggested by Slavicek [11].

## Conclusion

The dento-facial profile of Polish adolescents demonstrates specific characteristics which should be taken into account when diagnosing facial form in connection with orthodontic treatment planning in particular Polish patients.
